# The “Family Health = Family Wealth” intervention: study protocol for a pilot quasi-experimental controlled trial of a multi-level, community-based family planning intervention for couples in rural Uganda

**DOI:** 10.1186/s40814-022-01226-6

**Published:** 2022-12-24

**Authors:** Katelyn M. Sileo, Christine Muhumuza, Samuel Sekamatte, Haruna Lule, Rhoda K. Wanyenze, Trace S. Kershaw, Susan M. Kiene

**Affiliations:** 1 Department of Public Health, The University of Texas at San Antonio, One UTSA Circle, San Antonio, TX 78249 USA; 2 Department of Epidemiology and Biostatistics, Makerere University School of Public Health, Kampala, Uganda; 3 Gombe Hospital, Butambala District, Gombe, Uganda; 4 Global Centre of Excellence in Health (GLoCEH), Kampala, Uganda; 5 United States Agency for International Development (USAID), Kampala, Uganda; 6 Department of Disease Control and Environmental Health, Makerere University School of Public Health, Kampala, Uganda; 7 Division of Epidemiology and Biostatistics, San Diego State University School of Public Health, San Diego, CA USA; 8 Department of Social and Behavioral Sciences, Yale School of Public Health, New Haven, CT USA

**Keywords:** Couples, Contraception, Evaluation, Family planning, Uganda

## Abstract

**Background:**

Uganda has one of the highest fertility rates globally, but only 30% of women report using an effective method of contraception. Community-based, multi-level interventions are needed to help couples in rural Uganda overcome barriers to contraceptive use.

**Methods:**

This study will pilot test the Family Health = Family Wealth intervention, a multi-level, community-based intervention employing transformative community dialogues, which use facilitated discussion to reshape community norms that influence family planning acceptance, to alter individual attitudes and the perception of community norms that discourage family planning. Community dialogues are delivered to groups of couples over 4 sessions (two gender-segregated and two gender-mixed). Sessions simultaneously address individual and interpersonal-level determinants of family planning and link couples to family planning services. At the health system level, a refresher training will be conducted with health workers in the intervention community’s health center to address gaps in contraceptive knowledge and skills as identified from a needs assessment.

The intervention will be evaluated through a pilot quasi-experimental trial paired with a mixed methods process evaluation. Participants include 70 couples (*N*=140) randomized by community to the Family Health = Family Wealth intervention (*n*=35 couples) or to an attention-matched water, sanitation, and hygiene (WASH) intervention (*n*=35 couples). Participants include sexually active, married couples who are age 18 (or an emancipated minor) to 40 for women and age 18 (or an emancipated minor) to 50 for men, not pregnant, at least one person in the couple reports wanting to avoid pregnancy for at least a year, and not currently using a method of contraception or using a low-efficacy or ineffective method of contraception. The primary aims of the study are to (1) assess the feasibility of the intervention trial procedures, (2) the acceptability and feasibility of the intervention content and structure, and (3) explore the intervention’s preliminary effectiveness at increasing contraceptive use and affecting related outcomes among couples.

**Discussion:**

Filling the unmet need for family planning has important public health implications, including reductions in pregnancy-related health risks and deaths, and infant mortality. This pilot intervention trial will gather preliminary evidence on the acceptability, feasibility, and potential effect of a novel, multi-level, community-based intervention to increase contraceptive use among couples with an unmet need for family planning in rural Uganda. We aim to use the findings of this pilot study to refine the trial procedures and intervention content for a future, larger cluster randomized controlled trial to establish the intervention’s efficacy.

**Trial registration:**

ClinicalTrials.gov NCT04262882; registered on February 10, 2020.

## Background

Family planning through modern contraceptive methods has significant health benefits for women and children, including the prevention of pregnancy-related health risks and deaths, reductions in infant mortality and the rate of unsafe abortions, and the prevention of HIV transmission from mother-to-child and between partners (when condoms are used) [1]. Uganda made an ambitious commitment to the Family Planning 2020 (FP2020) initiative to reduce the nation’s unmet need for family planning to 10% by 2020 and recommitted to this goal for 2030 (FP2030) [2]. However, in 2020, the country was far from meeting this goal; 30.5% of married women had an unmet need for modern contraceptives [3]. Moreover, 41% of all pregnancies are unplanned [4], and Uganda had the seventh highest fertility rate in the world in 2021 at 5.45 children per woman [5], which is especially high in rural areas [4]. With a maternal mortality rate of 375 deaths per 100,000 live births compared to 19 deaths per 100,000 live births in the USA [6], it is estimated that if all women in need of contraceptives in Uganda were using them, maternal deaths could be reduced by 40% [7].

Uganda’s FP02030 efforts must be paired with interventions that generate demand for such services to optimize their reach. Misinformation and fear of side-effects [8–11], partner and peer influence [12–14], and cultural norms that promote large family size and traditional gender roles [15–18] negatively influence contraceptive use, and shape inequity in reproductive decision-making and communication between partners [19, 20]. However, despite numerous calls for multilevel interventions to address family planning needs, few interventions have incorporated this approach [21].

Community dialogues are a promising strategy to promote change at the individual, interpersonal, and community level [22]. Community dialogues follow a defined process to identify local drivers of sexual and reproductive health with community groups [22] and engage the community in problem-solving towards a common issue through community-based participatory methodologies [23]. The dialogue that takes place allows community members to critically think about social norms underpinning a community problem [24] and reconstruct community norms together, creating social environments that promote healthy behavior [25]. For reproductive health, community dialogues have been widely used by multinational agencies [22], but not rigorously tested and rarely published in peer-reviewed literature [26]. Successful examples demonstrate improvements in equitable relationships, community gender norms, and community ownership of a problem, but mainly focus on HIV and rely on qualitative methods [23, 27–34]. Thus, there is a gap of rigorous evaluations of community dialogues to support their effect on behavioral and health outcomes.

Community dialogues may be optimized when linked with other multi-level approaches [22], which may be needed to engage men and address relationship and community drivers of family planning. Emerging literature demonstrates interventions that engage male partners are more effective than those that do not [35, 36], but reviews of male engagement strategies conclude that evidence is still accumulating [37–40]. The following multi-level strategies have gained support, but are in need of further evaluation: tailoring messaging to men’s interests (e.g., economic benefits of family planning), improving partner communication and gender equitable attitudes, bringing services directly to communities, and demonstrating family planning support from community leaders and other men [19, 29, 38, 41–44].

This paper describes the protocol for the “Family Health = Family Wealth” pilot intervention, a multi-level, community-based intervention that engages groups of couples in transformative community dialogues, while addressing other multi-level barriers to contraceptive use, to increase contraceptive uptake/continuation and reduce the incidence of unintended pregnancy through improved intermediate outcomes (knowledge, attitudes, perceived community norms, communication, equity). Based on our preliminary research [45–47], the Family Health = Family Wealth intervention frames contraceptive use as one aspect of “family wealth” and emphasizes family planning’s economic and other family benefits. We test the acceptability, feasibility, and the preliminary effect of this intervention through a stage 1b pilot (i.e., feasibility and pilot testing of new behavioral interventions) quasi experimental trial with 70 couples. Following stage 1b pilot study guidelines [48, 49], the primary aims of our pilot trial are to assess acceptability and feasibility of the trial procedures and intervention content/structure, with the exploratory aim of assessing the intervention’s potential efficacy, detailed in the specific aims:

1. Assess the feasibility of trial procedures (i.e., community mobilization, recruitment, retention, outcome measurement) through the collection of process data and exit interviews with couples and group facilitators.
2. Assess the acceptability and feasibility of the Family Health = Family Wealth intervention content and structure through the collection of process data and exit interviews with couples and group facilitators.
3. Explore the potential effect of the Family Health = Family Wealth intervention on exploratory primary outcomes of contraceptive uptake and continuation and exploratory secondary outcomes (pregnancy incidence, knowledge, attitudes, perceived community norms, partner communication, and equity) among couples in the intervention community (relative to the attention-matched comparator community) through 6 months follow-up.

## Methods

### Setting

The study will be conducted in selected communities in a district in central Uganda, which is located 2 h from the capital of Kampala and has a population of approximately 150,000 in an area of approximately 270 mi^2^. Family planning services are provided for free at decentralized government public health facilities, integrated into general outpatient services. Local private not-for-profits (PNFPs) provided limited family planning services, while faith-based PNFP facilities promote natural methods only. In addition, some short-term methods can be purchased at local private shops. Within the district, government public health facilities range across five levels (I-IV) following the country’s decentralized health system structure. Health center IIs and above offer condoms, oral pills, and injectable contraceptives. Health center IIIs and above offer intrauterine devices (IUDs) and implants. Health center vs (general hospital) provide non-reversible methods (vasectomy, tubal ligation). The Village Health Team (VHT), a cadre of community health workers, serve as a liaison between the community and health facilities, and support community family planning efforts. VHTs provide community education about family planning and distribute short-term methods directly in the community (i.e., condoms, oral pills). In addition, an international nongovernmental organization, Marie Stopes, provides regular community outreach for contraceptive methods in selected villages within the district, including the provision of short- and long-term reversible methods.

### Study design

The study design is a pilot quasi-experimental controlled trial paired with a mixed methods process evaluation. The pilot quasi-experimental trial includes one community receiving the Family Health = Family Health intervention and one community receiving a time- and attention-matched water, sanitation, and hygiene (WASH) comparator intervention. The two communities selected in consultation with the District Health Team will be randomly assigned to receive the intervention or the comparator intervention, determined by coin toss. Seventy couples (*N*=140, 35 couples per community) will be recruited. We will conduct structured interviews at baseline (pre-intervention) and at 3- and 6-month follow-up.

**Fig. 1:**
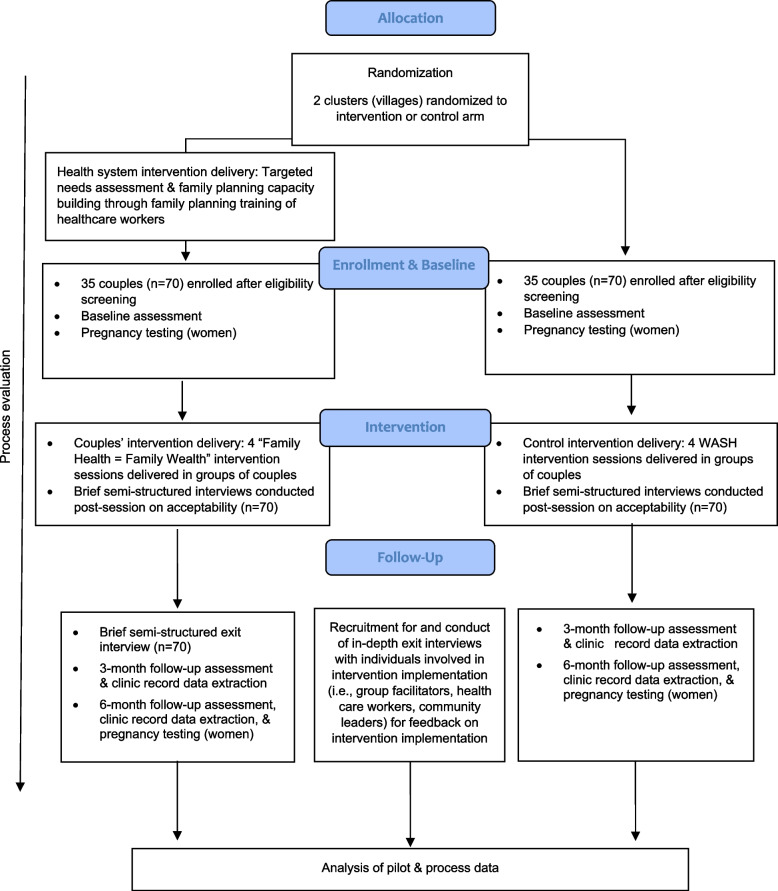
Consort diagram

In addition, mixed method data will be collected both during and after the trial to assess the acceptability and feasibility of the trial procedures and intervention content. Process data will be collected throughout the trial on community mobilization, participant retention, and other trial procedures. Brief, semi-structured interviews will be conducted with intervention and comparator participants during the trial immediately following each intervention session (*N*=140). Intervention fidelity will be monitored through direct observation of intervention sessions. Post-intervention, semi-structured exit interviews will be conducted with intervention participants (*N*=70), as well as individuals involved in the implementation of the intervention (i.e., group facilitators, health workers, community leaders). See Fig. 1 for the study design depicted in the CONSORT diagram, adapted for feasibility and pilot studies.

### Study population

The study population is adult couples, women aged 18 (or emancipated minors) to 40 and men aged 18 (or emancipated minors) to 50, residing in communities selected for inclusion in the study. The Ugandan National Council for Science and Technology defines emancipated minors as those under 18 who are married, having children, or are pregnant. Couples with an unmet need for family planning are the focus of the study, i.e., couples of reproductive age in which at least one person in the couple does not want to become pregnant within the next year but are not currently using modern contraceptives.

### Sampling and randomization

Two communities will be selected in partnership with the District Health Team. These communities will be matched by population (~2000), distance to a clinic with contraceptives, and demographics, randomly assigning one to receive the intervention and one as the comparator, determined by coin toss. If needed, the recruitment could expand to include nearby communities within the same geographic area. Communities will be at least 15 km in distance from one another and not located along the same road to avoid potential contamination. Snowball sampling will also be used to identify couples living within the sampled communities. Leveraging existing social networks through snowball sampling may enhance our intervention’s effect on social norms, as family, peers, and neighbors more directly affect contraceptive decisions than strangers.

### Procedures

#### Eligibility, recruitment, and informed consent procedures

After we randomly allocate the communities to the intervention and comparator arm, we will recruit couples with an unmet need for family planning from the selected communities for participation in our intervention trial. Thirty-five couples (70 individuals; 35 women, 35 men) will be recruited from each community (intervention and comparator intervention). In each community, VHTs will assist our research staff in identifying potentially eligible couples. For the couples that we attempt to recruit based on the VHT’s referral, the VHT will first independently visit couples’ homes to introduce them to the study and gain permission to return with the research assistant. VHTs will be blinded to the study’s purpose and will not participate in the screening of participant eligibility. If permission is granted, the VHT will accompany our research assistant to couples’ homes to introduce our research assistant and then leave the house. Participants in both conditions will receive the same recruitment script and consent form. Research assistants and participants in both conditions will be told that the study is focused on family health and wellness, with the intervention program including a range of topics affecting family wellness such as health, healthy relationship, water and sanitation hygiene, family planning, and economic wellness, with the content they receive chosen through random selection. This serves to blind participants and research assistants to the purpose of the intervention and reduce selection bias.

If both partners are together during this initial meeting, the research assistant will inform them of the overall study together, but will screen and assess eligibility separately, ensuring that participants are able to answer the eligibility screening questions about fertility preferences and current use of contraceptives in a private setting to ensure confidentiality. If only one partner is present, the research assistant will proceed with the eligibility screening and conduct the screening and all subsequent steps at a later date. See Table 1 for a summary for the eligibility criteria. No identifying information will be collected for eligibility screening. If eligible and interested, the research assistant will then obtain written informed consent from the participants. The interviewer will proceed to conduct the baseline interview after obtaining written informed consent for both partners.

**Table 1:** Summary of inclusion criteria for couple enrollment in the pilot intervention trial

1	Married or considers themselves married
2	Couple living together most of the time
3	Living within the selected communities
4	Age 18 (or an emancipated minor) to 40 for women; age 18 (or an emancipated minor) to 50 for men
5	Luganda speaking
6	Woman not currently pregnant (self-reported and then confirmed with a pregnancy test for those meeting all other criteria)
7	Has never been told by a doctor that they are infertile
8	At least one person in the couple reports wanting to avoid pregnancy for at least a year
9	Not using a method of contraception or using a low-efficacy or ineffective method of contraception^a^
10	Never used a non-reversible method (male or female sterilization)
11	Woman reporting having had sex in the past 3 months with spouse or planning to resume sex within the next 3 months with spouse if postpartum

^a^Low-efficacy or ineffective method of contraception defined as using condom less than 100% of the time, lactational amenorrhea, Fertility Awareness-Based methods (e.g., counting method), withdrawal method, spermicide, emergency contraception, and sponge

If an eligible participant is interested, but their partner declines participation, the research assistant will not proceed to recruit them in the study. However, they will provide a referral to local health services, including family planning services, for a nearby clinic to the interested participant (in both the intervention and comparator communities to maintain balance between the study arms).

After we successfully enroll the first few couples that meet eligibility criteria and consent to participation, we will then use snowball sampling to identify the next couple. That is, we will ask participants to refer another couple they know that lives within the sampled community of similar age that might also be interested in participating. The participating couple will be asked to refer their friends to our research staff or to the VHT, providing our contact information. We will wait for new couples to initiate contact with our study team and provide permission for our research assistant to visit their home before doing so. If we reach the end of a network, or if any couple is not able/willing to refer another couple, we will return to the VHT to identify a new couple, from which we will continue our snowball sampling procedures.

### Description of the study arms

#### Comparison arm: water, sanitation, and hygiene (WASH) intervention

Participants in the comparison arm will receive an attention-matched comparator intervention, including four community dialogue sessions focused on water, sanitation, and hygiene (WASH) led by a trained facilitator. The content will include directive education, open dialogue, and group problem-solving using an adapted version of an existing curriculum developed for community health workers in Ethiopia with support from USAID and the Water and Sanitation Program [50]. The focus of the content is community-led total sanitation, a widely adopted approach to WASH behavior change, focusing on an array of WASH behaviors (i.e., clean water, hand-washing, latrine use, personal and home hygiene, safe food preparation). Following the structure of our family planning intervention, comparator sessions will include two gender-segregated and two gender-mixed sessions delivered to groups of couples. We will also tailor the intervention to the local Ugandan context, guided by the study’s investigators from Uganda and with input from local VHTs and midwives. This intervention was chosen as a comparator because it was similar in time and structure (e.g., delivered in community groups by a trained facilitator) to the planned intervention, making it easy adaptable to be time and attention-matched. The topic of WASH was selected based on consultation with local stakeholders and community members, who gave feedback that the content would be of interest and deemed culturally appropriate to community members, which we considered important for retention purposes, without the content overlapping with family planning.

#### Family Health = Family Wealth intervention arm (see Table 2)

Before the implementation of the couples’ intervention, a needs assessment will be conducted at the local clinics to identify gaps in health worker capacity specific to the provision of contraceptive methods and family planning education. In partnership with the District Health Team, a targeted refresher training will be conducted with health workers in the intervention community’s health center to address gaps in knowledge and skills.

**Table 2:** Intervention arm: multilevel, community-based intervention

	**Health-system strengthening components**
Pre-intervention health worker capacity building	• Needs assessment conducted at public health facilities in intervention village to assess gaps in contraceptive knowledge and skills among health workers. • Tailored family planning refresher training provided in partnership with the District Health Team to address training gaps.
	**Couples’ intervention session core components**
Session 1 Gender-segregated, 180 min	• Guided discussion to identify gender-specific definitions of “family wealth,” interpersonal and community barriers to family health and wealth, and redefine group norms on a “successful” family (men and women’s groups)
Session 2 Gender-segregated, 180 min	• Physical health: contraceptive education with a midwife (women’s groups) • Relationship health: discussion on healthy relationships and family planning (partner violence, communication, decision-making, caregiver roles, gender norms); role modeling of gender equitable couples (men’s groups) • Economic health: business skill training with a local business expert (men and women’s groups)
Session 3 Gender-mixed, 180 min	• Physical health: contraceptive education from a midwife; provide family planning/linkages to care; create a “Family Action Plan”—setting family size and contraception goals • Relationship health: communication skills building activities; create a Family Action Plan—setting relationship goals (take home assignment) • Economic health: family budgeting
Session 4 Gender-mixed, 180 min	• Relationship health: communication skills building activity • Revisit Family Action Plan goals as a couple • Guided discussion to identify community barriers and solutions for family planning access/uptake • Introduction to a “Community Action Plan” • Provide family planning/linkages to care

Participants in the intervention community will receive the Family Health = Family Wealth intervention consisting of four sessions: two gender-segregated and two gender-mixed. The intervention content was informed by preliminary research with women and men from the same study area [45–47], and a more recent qualitative needs assessment conducted as the precursor to this pilot trial that identified individual, interpersonal, community, and health system-level drivers of contraceptive use, guided by the social ecological model [51–53]. Each of the 4 sessions include community dialogues, which are grounded in Campbell and Cornish’s social psychological theory of transformative communication [54], the primary mechanism of action to affect change across the individual, interpersonal, and community levels, specifically through change in individual attitudes, interpersonal communication, and the perception of community norms related to family planning acceptance and gender equity. Our facilitated discussions will focus on community norms around gender roles, equity, and family size and critically analyze the social and community influences of “family-wealth” and poverty with the overall goal of reconstructing individual attitudes and group norms on paths to/definitions of a “successful family” inclusive of family planning.

Dialogues are enhanced in sessions 2 and 3 to address knowledge, motivation, self-efficacy, and relationship dynamics, tailored to men and women. Based on our preliminary research on gender-specific family planning facilitators [45–47], the overarching theme of each session is “Family Health = Family Wealth.” We conceptualize “family wealth” as being made up of relationship, economic, and physical well-being and deliver content to demonstrate how family planning contributes to family health and wealth in each area. The inclusion of community leaders to endorse behavior change in group dialogues can increase success of transformative communication and facilitate the allocation of local resources to implement group-generated action plans [22, 31, 33]. Therefore, we will select and train both female and male community leaders to endorse family planning at the community-level at the beginning and end of the program, involve local business experts to engage men and women in economic skills training, and involve midwives and VHTs directly in program delivery. It is common in community dialogues for the group to work together to develop a “Community Action Plan” to elicit community-derived solutions that utilize existing resources and increase community ownership of these solutions [26]. We adapt this concept into “Family Action Plans” for couples in session 3 and introduce “Community Action Plans” in session 4. At the end of sessions 3 and 4, participants will have the opportunity to receive short-term contraceptive methods directly, or referrals for long-term methods, and family planning couples counseling from a midwife and/or VHT.

### Data collection procedures and measures

#### Feasibility aim 1 (trial procedures)

We will develop process monitoring data collection tools to capture process data on the feasibility of trial procedures, or the extent to which these procedures can be successfully carried out in this setting [55], specific to community mobilization, recruitment, retention, and the assessment process, which will be triangulated/explained with qualitative exit interviews and direct observation when appropriate. All intervention sessions will be audio-recorded and a randomly selected portion (~20%) of the dialogues transcribed and analyzed for fidelity following a constructed checklist. The measurement of specific outcomes is detailed below:

- *Community mobilization*: Percent of households that accepted the invitation to learn more about the study and collected through process data/study records.
- *Recruitment*: Number of couples enrolled per month and the percent of eligible couples where one partner declined, and reasons for decline by gender, collected through process data/study records.
- *Retention*: Treatment-specific retention rates through 6-month follow-up and reason for drop-out by gender, collected through observation and exit interviews with participants.
- *Assessment process*: Percent of planned assessments completed and duration of assessments collected through process data collection; barriers to pregnancy testing and the delivery of pregnancy test results gathered through exit interviews with participants and research staff.
- *Fidelity*: Degree to which intervention was implemented as prescribed in the protocol, measured through fidelity check-lists completed by intervention facilitators, direct observation by research staff, and the audio-recording and analysis of randomly selected sessions (as previously described).

#### Feasibility/acceptability aim 2 (intervention content/structure)

After each intervention and comparator session, all individuals will complete a brief semi-structured interview (~5 min) to assess acceptability of the intervention content and structure, rating perceived importance and satisfaction with intervention content, and an open-ended question on what they liked and disliked. These brief semi-structured interviews will also include questions to assess any unanticipated negative adverse events occurring because of the intervention (e.g., partner conflict). Interviews will be conducted immediately following each intervention session by the intervention facilitators and by one of the study’s Primary Investigators.

After all intervention sessions are completed, semi-structured exit interviews (~15 min) will be conducted with intervention participants (*n*=70). These interviews will be conducted by a trained qualitative interviewer, not otherwise involved in the intervention delivery or outcome assessment, within a 3-week participant completion of the last intervention session. They will be conducted over the phone or in-person at a place convenient to the participant. The aim of the exit interviews is to further assess acceptability, feasibility, appropriateness, areas for improvement, and progress on “Family Action Plans” among participants.

After the completion of the intervention sessions, semi-structured interviews will also be conducted with individuals involved in intervention implementation include the trained group discussion leaders (*n*=2), health workers, and VHTs that will deliver family planning education and contraceptive methods (*n*~2), and trained community leaders and local business experts that will co-facilitate discussions (*n*~2–4). The aim of the exit interviews with intervention facilitators is to assess acceptability and feasibility of the intervention and involvement in community leaders/health workers in the intervention, barriers to implementation, and areas for improvement from the perspectives of the facilitators. Exit interviews will be conducted in-person or over the phone within the 6 months following the completion of the last intervention session by a third-party, trained qualitative interviewer or one of the study’s Primary Investigators.

The measurement of the primary outcomes for the feasibility/acceptability of the intervention gathered from these data are detailed as follows:

- *Feasibility*: Whether the intervention can be carried out and utilized, and if it can linked to family planning services, including whether contraceptives can be distributed directly during sessions, collected through observation and exit interviews with participants and individuals involved in the implementation of the intervention (i.e., group facilitators, health workers, community leaders) [55]
- *Acceptability*: Satisfaction with the intervention, collected through brief semi-structured intervention after each intervention session and post-intervention exit interviews with participants [55]
- *Perceived fit*: Appropriateness for setting and to improve care quality and patient outcomes, collected through observation and exit interviews with participants and individuals involved in the implementation of the intervention (i.e., group facilitators, health workers, community leaders) [55]
- *Perceived barriers*: Perceived difficulty of and barriers to implementation and utilization collected through observation, brief semi-structured intervention after each intervention session, and exit interviews with participants and individuals involved in the implementation of the intervention (i.e., group facilitators, health workers, community leaders)

#### Exploratory aim 3 (intervention effect)

At baseline, immediately following enrollment, and at 3- and 6-month follow-up, research assistants will collect demographic information (baseline only) and measures to assess family planning determinants in a one-on-one interview. Data will be collected using a computerized structured questionnaire. All couples will be interviewed separately; the interview will take place in a private setting within the participant’s home, another agreed upon location, or over the phone if necessary. At baseline and 6 months, research assistants will also collect pregnancy test results from women, following a urine Human Chorionic Gonadotropin rapid pregnancy test. Finally, at 3- and 6-month follow-up, research assistants will go to the local health facility to extract data from medical charts on participants’ use of contraceptive methods in the prior 3 months to triangulate self-report. Pregnancy test results and clinic record data will be double entered into an electronic data collection form.

While this pilot trial is not powered to detect statistically significant changes in all of these outcomes, the collection of the full set of exploratory outcomes helps to achieve aim 2, the pilot testing of intervention trial procedures, including the development of data collection tools, procedures, and data on their feasibility. For example, piloting the administration of pregnancy tests and procedures for delivering the results to couples will allow us to gather information to inform procedures appropriate for the local context.

The outcomes, how they are measured/collected, and timeframes of collection are displayed in Table 3.

**Table 3:** Exploratory primary outcome measures used to pilot the acceptability and feasibility of measurement tools and procedures, and the intervention’s preliminary effect on family planning outcomes

**Exploratory primary outcome measures**	**Data collection procedures/measures**	**Time frame**
*Modern contraceptive use/continuation*	Self-reported measures for current contraceptive use are adapted from Uganda Demographic and Health Survey (DHS) measures [4]. Defined as the usage of pills, injection, condoms (self-report, ≥90% of sex acts), IUD, implant, tubal ligation, vasectomy, measured through women’s clinic records (men’s for vasectomy) and triangulated with self-report [56, 57]	3 and 6 months
***Exploratory secondary outcome measures***	**Data collection procedures/measures**	**Time frame**
*Pregnancy incidence and unintended pregnancy incidence*	Measured by urine Human Chorionic Gonadotropin rapid pregnancy tests. At the baseline and 6-month home visit, women will be instructed to take the pregnancy test by the research assistant	Baseline and 6 months
*Family planning intentions*	Items adapted from the Uganda DHS on intentions to use contraceptives and family planning services in the future [4]	Baseline, 3 and 6 months
*Knowledge of contraceptives*	Items from the Uganda DHS measures that assess the participants’ awareness of different contraceptive methods [4]	Baseline, 3 and 6 months
*Family planning attitudes*	Scale developed for use in Uganda and found reliable and predictive of contraceptive use in Uganda in our prior research (*α* = 0.80) [47] to measure how participants would feel about using contraceptive methods	Baseline, 3 and 6 months
*Family planning norms*	Items adapted from the Family Planning Approval Index to assess the perceived acceptance of family planning and contraceptive use among partner, family, peers, and broader community [58]	Baseline, 3 and 6 months
*Fertility desire*	Measured using an item from the Uganda DHS item on the participants’ desired number of children [4]	Baseline, 3 and 6 months
*Fertility concordance between partners*	Measured by the difference between a couples reported ideal family size using items from the Uganda DHS on the participants’ desired number of children [4].	Baseline, 3 and 6 months
*Partner communication about family planning*	Items on the frequency of communication with partner about family planning and contraceptive use constructed for our prior studies in Uganda [45, 47]	Baseline, 3 and 6 months
*Gender equitable attitudes*	The Gender Equitable Men scale [59], which measures participants’ endorsement of traditional gender norms and attitudes on gender equity validated in Tanzania and Ghana [60], with good reliability in African settings (Cronbach’s *α*=0.79–0.88) [60–63]	Baseline, 3 and 6 months

#### Sample size justification

As an exploratory pilot, we base our sample size for our quasi-experimental controlled trial on guidelines for Stage 1b studies (feasibility and pilot testing of new behavioral interventions), which suggest 15–30 participants per cell [49], choosing 35 couples per condition (*n*=70 individuals per condition). Even assuming moderate attrition (20%), we would have 28 couples per condition, which is still within the guidelines for pilot studies.

### Data analysis approach

Quantitative process data and data collected from brief semi-structured questionnaires intervention session will be analyzed using frequencies and descriptive statistics (e.g., counts, percents, means and standard deviations, or medians and inter-quartile ranges).

Qualitative data collected through exit interviews will be audio-recorded, translated, transcribed, and analyzed using a thematic analysis approach [64]. The investigative team will review the transcripts to identify potential themes and develop a coding scheme guided by the constructs outlined in Table 3. Two trained research assistants will independently code the data, which will be reviewed by the Primary Investigators. The team will review codes for consistency, refine the coding scheme as needed, and identify major themes, repeating this process until consensus is reached. Following Creswell and Plano-Clark, qualitative findings will be mixed with quantitative process data as appropriate during the presentation and interpretation of results [65].

As a pilot intervention trial, the study’s exploratory aim to gather preliminary effects of the intervention on family planning outcomes will be achieved through mainly descriptive analyses. Using SPSS v.28, we will use frequencies to examine the total uptake of contraceptives (using an effective method, yes vs. no) in the intervention and comparator arms and will use frequencies and descriptive statistics and measures of variance to describe the study’s exploratory secondary outcomes across the two study arms. We will look at the percent difference between contraceptive uptake between the two arms and use the magnitude of the difference (if present).

### Progression criteria

Progression criteria to help determine the next step beyond this pilot trial are detailed in Table 4. Following Thabane et al.’s recommendations [66], the progression criteria will be used to determine whether the next step is progression to a larger, fully powered cluster randomized controlled trial without modification to the protocol, progression without modifications but with close monitoring, progression with modifications, or no progression if deemed not feasible/acceptable even after modifications. The progression criteria focus on the key outcomes related to the acceptability of the intervention, feasibility of recruitment, fidelity of intervention implementation, and feasibility of outcome measurement.

**Table 4:** Progression criteria for the Family Health = Family Wealth pilot intervention

Outcomes	Criteria	No modifications	Consider modification and closely monitor	Modifications needed to progress	Stop (not feasible/acceptable)
**Acceptability of intervention**	% of participants reporting being satisfied or very satisfied with the intervention sessions	90–100%	80–89%	50–89%	<50%
**Feasibility of recruitment**	% of eligible participants (couples) that can be recruited	75–100%	70–74%	50–69%	<50%
**Fidelity of intervention**	% of all intervention sessions delivered to participants	89–100%	72–88%	50–71%	<50%
**Feasibility of outcome measurement**	% complete follow-up in recruited participants	91–100%	79–90%	60–78%	<60%

Following Thabane et al., [66] the information above will be used to determine one of the following outcomes for the pilot study:

(i) Stop—main study not feasible/acceptable

(ii) Continue, but modify protocol—feasible/acceptable with modifications

(iii) Continue without modifications, but monitor closely and consider modifications to improve protocol—feasible/acceptable with close monitoring; modifications may improve feasibility/acceptability

(iv) Continue without modifications—feasible/acceptable as is

### Ethics

The study has obtained approval of all study procedures from the institutional review boards at the University of Texas at San Antonio (protocol # 19-253) and the Makerere University School of Public Health (protocol # 748). The Ugandan National Council for Science and Technology has also granted study approval. Mobilization meetings were also conducted to garner district buy-in, and entry into the communities was permitted by district leadership. We will obtain written informed consent for participation in the pilot intervention trial from each person in the couple separately. We will also obtain written informed consent for participation in exit interviews from all individuals involved in intervention implementation (i.e., group facilitators, health workers, VHTs, community leaders). In addition, a data safety monitoring board (DSMB) will be assembled to provide third party review of the study protocol before implementation, and review data related to participant safety every 3 months throughout the duration of data collection activities. We expect that the overall risk to benefit ratio will be favorable to individuals participating in the study. The main potential risks of the study are summarized in Table 5, with details on strategies that will be employed to minimize each risk.

**Table 5:** Overview of ethical considerations: potential risks of participation and planned strategies for risk mitigation

Risks	Safeguards for risk mitigation
**Potential for breaches in confidentiality related to collected data**	(1) The use of unique identifiers instead of medical identification/record numbers or participant names (2) The storing of the lists that link the participants to their unique identifiers in locked, secure locations (3) The use password protection for all data collected and/or stored electronically (4) Training all study staff in the importance of and procedures for protecting participants’ confidentiality
**Potential to experience discomfort while discussing sensitive information during interviewer administered computerized questionnaires and group intervention sessions**	(1) IRB-approved consent forms to convey that the survey portion of the research project and group sessions involve sensitive topics (2) The ability to skip any questions that makes one uncomfortable and to withdraw from the study any time (3) Training all study staff in the importance of procedures for protecting participants’ confidentiality (4) Training of study staff to approach sensitive topics in a culturally appropriate and non-judgmental way
**Potential unintended negative consequences on participants in the intervention (e.g., conflict between partners)**	(1) Inform participants of potential risks in the informed consent process and discourage participation among individuals who fear participation could increase their risk for violence victimization. (2) Ask participants to inform study staff throughout the study if they feel they are experiencing an increased risk of violence so that they can be linked to resources and removed from the study if deemed necessary (3) Monitor increased risk of violence through the post-intervention interview (4) Monitor increased risk of violence through the 3-month and 6-month questionnaires (5) Training of group facilitators to recognize disagreement between couples and will provide counseling to couples to minimize discordance, and the occurrence of conflict (6) Training of group facilitators to guide couples’ in selecting action plan items that are appropriate for their circumstances

## Discussion

This protocol describes the pilot implementation and evaluation of a multi-level, community-based family planning intervention for couples in rural Uganda. Couples receiving the Family Health = Family Wealth intervention will participate in multiple group sessions (2 gender-segregated, 2 gender-mixed) aimed to address multi-level barriers to contraceptive use, including community dialogues with groups of couples to reconstruct group norms enhanced with activities to improve knowledge, motivation, couple dynamics, and link couples to services. The intervention also aims to reduce health system barriers to contraceptive use through targeted capacity building for family planning through the training of healthcare workers in the intervention community and through the direct distribution of short-term contraceptive methods during group sessions. This pilot study will indicate if the trial procedures and intervention content are acceptable and feasible and provide preliminary evidence of whether couples in Family Health = Family Wealth intervention show trends towards greater contraceptive uptake/continuation and improvement in intermediate outcomes (knowledge, attitudes, perceived norms, partner communication, equity) at 6 months compared to couples receiving a time- and attention-match WASH comparator intervention.

This pilot intervention study has the potential to advance science and improve public health service delivery in several ways. This study will fill several gaps in the family planning intervention literature, where there is a lack of rigorously tested family planning interventions that take a multi-level approach, few interventions that show effects beyond knowledge and attitudes (i.e., on contraceptive use and pregnancy incidence), and a need for more research on strategies to engage male partners [21, 36, 67–70]. Those in the intervention arm may benefit from transformative dialogues that shift individual and community attitudes towards more gender equitable attitudes and new definitions of a “successful” family inclusive of family planning. The intervention can create a social environment supportive of family planning and gender equity, which may subsequently influence perceived social acceptance of family planning and encourage greater use of family planning services. Secondary benefits of promoting gender equitable attitudes through our intervention may include the potential for reductions in intimate partner violence, improvements in women’s empowerment in relationships and in the broader community through participation in interactive group forums, and greater engagement among men in family planning and health services more generally.

Importantly, family planning through modern contraceptive methods has significant health benefits for women and children [1]. Family planning also has economic benefits for families and for society, especially in under-resourced settings. Couples who space and limit their pregnancies have more earning potential and families can devote more resources to each child. On a population-level, family planning is critical to achieving the “demographic dividend” in low-income nations, a window of economic growth resulting from decreased fertility rates and subsequent increases in the proportion of working-age people relative to dependent children.

### Limitations

We chose to randomize by community, and not by couple, because intervention components are delivered at the community level. As a pilot trial, we can only randomize two communities, which could introduce confounding variables given too few clusters. However, this pilot can inform the procedures for a larger CRCT after acceptability and feasibility are established. To reduce confounding variables, we will attempt to match intervention and comparator communities on demographics. Another potential limitation includes the potential for contamination between treatment conditions. This is mitigated by choosing communities at least 15 km apart and not along a main road. We will blind participants and interviewers to study condition. While un-blinding is possible, we will mitigate the risk of un-blinding by presenting the overall program as a family health and wellness intervention that includes a range of topics related to economic health, relationship health, and physical health, including both family planning and WASH topics. Participants and interviewers will be told that participants are randomized to receive some, all, or none of this content in order to attempt to mask that the true goal of the study is to increase contraceptive use. Finally, we include pregnancy incidence as an exploratory secondary outcome, informing the acceptability of pregnancy testing procedures of a future trial. While we have limited power to detect differences in pregnancy incidence, trends towards change in contraceptive use and related determinants will provide evidence to support a future, fully powered trial, the feasibility of which is strengthened by piloting pregnancy testing and other trial procedures.

## Conclusion

The pilot trial for the Family Health = Family Wealth intervention will provide data on acceptability, feasibility, and preliminary effects on contraceptive use and exploratory secondary outcomes among couples with an unmet need for family planning. If progressed to a future trial, this trial will assess the intervention’s effects on contraceptive use, unintended pregnancy, and potentially the feasibility of using the intervention as a platform for community distribution of contraceptives. Thus, this intervention has the potential to be sustained through Uganda’s national scale up of community-based family planning, which will require the simultaneous implementation of evidence-based, demand-generation activities to increase community acceptance of family planning. This intervention also has the potential to be generalizable to other African and South East Asian countries, where high unmet need for contraceptives is similarly tied to gender norms, relationship equity, and community dynamics and where community-based health service models are utilized [8, 71–73].

## Data Availability

Data sharing not applicable for this protocol article as no datasets were generated or analyzed.

## References

[1] World Health Organization (WHO). Family planning/contraception: fact sheet. 2018. Retrieved from http://www.who.int/mediacentre/factsheets/fs351/en/.

[2] The Government of Uganda. Family planning 2020 commitment: Govt. of Uganda. Family Planning Summit in London, UK: FP2020; 2017. Available from: http://www.familyplanning2020.org/entities/80.

[3] FP2020. Uganda - FP2020 Core Indicator Summary Sheet: 2018-2019 Annual Progress Report. 2020. Available from: http://www.familyplanning2020.org/sites/default/files/Data-Hub/2019CI/Uganda_2019_CI_Handout.pdf.

[4] Uganda Bureau of Statistics (UBOS), ICF. Uganda Demographic and Health Survey 2016. Kampala, Uganda and Rockville, Maryland, USA: UBOS and ICF; 2018.

[5] Central Intelligence Agency (CIA). The World Factbook. Country comparison: total fertility rate. 2021. Retrieved from: https://www.cia.gov/the-world-factbook/field/total-fertility-rate/country-comparison.

[6] Central Intelligence Agency (CIA). The World Factbook. Country comparison: maternal mortality rate. 2017. Retrieved from: https://www.cia.gov/the-world-factbook/field/total-fertility-rate/country-comparison.

[7] Guttmacher Institute (2009). Benefits of meeting contraceptive needs of Ugandan women.

[8] Wulifan JK, Brenner S, Jahn A, De Allegri M (2016). A scoping review on determinants of unmet need for family planning among women of reproductive age in low and middle income countries. BMC Womens Health.

[9] Wanyenze RK, Wagner GJ, Tumwesigye NM, Nannyonga M, Wabwire-Mangen F, Kamya MR (2013). Fertility and contraceptive decision-making and support for HIV infected individuals: client and provider experiences and perceptions at two HIV clinics in Uganda. BMC Public Health.

[10] Thummalachetty N, Mathur S, Mullinax M, DeCosta K, Nakyanjo N, Lutalo T (2017). Contraceptive knowledge, perceptions, and concerns among men in Uganda. BMC Public Health.

[11] Kabagenyi A, Jennings L, Reid A, Nalwadda G, Ntozi J, Atuyambe L (2014). Barriers to male involvement in contraceptive uptake and reproductive health services: a qualitative study of men and women’s perceptions in two rural districts in Uganda. Reprod Health.

[12] Prata N, Bell S, Fraser A, Carvalho A, Neves I, Nieto-Andrade B (2017). Partner support for family planning and modern contraceptive use in Luanda, Angola. Afr J Reprod Health.

[13] Aransiola JO, Akinyemi AI, Fatusi AO (2014). Women’s perceptions and reflections of male partners and couple dynamics in family planning adoption in selected urban slums in Nigeria: a qualitative exploration. BMC Public Health.

[14] Heck CJ, Grilo SA, Song X, Lutalo T, Nakyanjo N, Santelli JS. “It is my business”: a mixed-methods analysis of covert contraceptive use among women in Rakai, Uganda. Contraception. 2018. 10.1016/j.contraception.2018.02.017 PMC6041694 29514043

[15] Mutumba M, Wekesa E, Stephenson R (2018). Community influences on modern contraceptive use among young women in low and middle-income countries: a cross-sectional multi-country analysis. BMC Public Health.

[16] Stephenson R, Baschieri A, Clements S, Hennink M, Madise N (2007). Contextual influences on modern contraceptive use in sub-Saharan Africa. Am J Public Health.

[17] Ghanotakis E, Hoke T, Wilcher R, Field S, Mercer S, Bobrow EA (2017). Evaluation of a male engagement intervention to transform gender norms and improve family planning and HIV service uptake in Kabale, Uganda. Global Public Health.

[18] Kabagenyi A, Reid A, Ntozi J, Atuyambe L (2016). Socio-cultural inhibitors to use of modern contraceptive techniques in rural Uganda: a qualitative study. Pan Afr Med J.

[19] Hartmann M, Gilles K, Shattuck D, Kerner B, Guest G (2012). Changes in couples’ communication as a result of a male-involvement family planning intervention. J Health Commun.

[20] Paek HJ, Lee B, Salmon CT, Witte K (2008). The contextual effects of gender norms, communication, and social capital on family planning behaviors in Uganda: a multilevel approach. Health Educ Behav.

[21] Scholmerich VL, Kawachi I (2016). Translating the social-ecological perspective into multilevel interventions for family planning: how far are we?. Health Educ Behav.

[22] High-Impact Practices in Family Planning (HIPs). Community group engagement: changing norms to improve sexual and reproductive health. Washington, DC: USAID; 2016. Available from: https://www.fphighimpactpractices.org/wp-content/uploads/2018/03/CommunityGroupEngagement.pdf.

[23] UNDP (2004). Upscaling community conversations in Ethiopia: unleashing capacities of communities for the HIV/AIDS response.

[24] Freire P (1973). Education for critical consciousness.

[25] Tawil O, Verster A, O’Reilly KR (1995). Enabling approaches for HIV/AIDS prevention: can we modify the environment and minimize the risk?. AIDS..

[26] Campbell C, Nhamo M, Scott K, Madanhire C, Nyamukapa C, Skovdal M (2013). The role of community conversations in facilitating local HIV competence: case study from rural Zimbabwe. BMC Public Health.

[27] Underwood C, Brown J, Sherard D, Tushabe B, Abdur-Rahman A (2011). Reconstructing gender norms through ritual communication: a study of African transformation. J Commun.

[28] Figueroa ME, Poppe P, Carrasco M, Pinho MD, Massingue F, Tanque M (2016). Effectiveness of community dialogue in changing gender and sexual norms for HIV prevention: evaluation of the Tchova Tchova program in Mozambique. J Health Commun.

[29] Schuler SR, Nanda G, Ramirez LF, Chen M (2015). Interactive workshops to promote gender equity and family planning in rural Guatemalan communities: results of a community randomized trial. J Biosoc Sci.

[30] Tesfaye AM. Using community conversation in the fight against HIV and AIDS. Journal of Development and Communication. Studies. 2013:2(2-3).

[31] Campbell C, Scott K, Nhamo M, Nyamukapa C, Madanhire C, Skovdal M (2013). Social capital and HIV competent communities: the role of community groups in managing HIV/AIDS in rural Zimbabwe. AIDS Care.

[32] Campbell C, Nair Y, Maimane S, Sibiya Z (2008). Supporting people with AIDS and their carers in rural South Africa: possibilities and challenges. Health Place.

[33] Mutale W, Masoso C, Mwanza B, Chirwa C, Mwaba L, Siwale Z (2017). Exploring community participation in project design: application of the community conversation approach to improve maternal and newborn health in Zambia. BMC Public Health.

[34] Women UN, M. (2013). Advancing gender equality: promising practices, case studies from the millennium development goals achievement fund. Application of the Community Conversation Enhancement Methodology for Gender Equality in Namibia.

[35] Phiri M, King R, Newell JN (2015). Behaviour change techniques and contraceptive use in low and middle income countries: a review. Reprod Health.

[36] Belaid L, Dumont A, Chaillet N, Zertal A, Brouwere VD, Hounton S (2016). Effectiveness of demand generation interventions on use of modern contraceptives in low- and middle-income countries. Tropical Med Int Health.

[37] Institute for Reproductive Health (2014). Male engagement in family planning: reducing unmet need for family planning by addressing gender norms.

[38] High-Impact Practices in Family Planning (HIPs) (2016). Engaging men and boys in family planning: a strategic planning guide.

[39] Tokhi M, Comrie-Thomson L, Davis J, Portela A, Chersich M, Luchters S (2018). Involving men to improve maternal and newborn health: a systematic review of the effectiveness of interventions. PLoS One.

[40] Barker G, Ricardo C, Nascimento M (2007). Engaging men and boys in changing gender based inequity in health: evidence from programme interventions.

[41] Shattuck D, Kerner B, Gilles K, Hartmann M, Ng'ombe T, Guest G (2011). Encouraging contraceptive uptake by motivating men to communicate about family planning: the Malawi male motivator project. Am J Public Health.

[42] MacDonald L, Jones L, Thomas P, Thu L, FitzGerald S, Efroymson D (2013). Promoting male involvement in family planning in Vietnam and India: HealthBridge experience. Gend Dev.

[43] Avogo W, Agadjanian V (2008). Men’s social networks and contraception in Ghana. J Biosoc Sci.

[44] Doyle K, Levtov RG, Barker G, Bastian GG, Bingenheimer JB, Kazimbaya S (2018). Gender-transformative Bandebereho couples’ intervention to promote male engagement in reproductive and maternal health and violence prevention in Rwanda: findings from a randomized controlled trial. PLoS One.

[45] Sileo KM, Wanyenze RK, Lule H, Kiene SM (2015). Determinants of family planning service uptake and use of contraceptives among postpartum women in rural Uganda. Int J Public Health.

[46] Sileo KM, Wanyenze RK, Lule H, Kiene SM (2017). “That would be good but most men are afraid of coming to the clinic”: men and women’s perspectives on strategies to increase male involvement in women’s reproductive health services in rural Uganda. Int J Public Health.

[47] Kiene SM, Hopwood S, Lule H, Wanyenze RK (2014). An empirical test of the theory of planned behaviour applied to contraceptive use in rural Uganda. J Health Psychol.

[48] Leon AC, Davis LL, Kraemer HC (2011). The role and interpretation of pilot studies in clinical research. J Psychiatr Res.

[49] Rounsaville BJ, Carroll KM, Onken LS (2001). A stage model of behavioral therapies research: getting started and moving on from stage I. Clin Psychol Sci Pract.

[50] Amhara National Regional Sate Health Bureau (2013). Training manual on hygiene and sanitation promotion and community mobilization for volunteer community health promotors (VCHP). Amhara Regional Health Bureau.

[51] Brofenbrenner U (1977). Toward an experimental ecology of human development. Am Psychol.

[52] McLeroy KR, BD, Steckler A, Glanz KA (1988). An ecological perspective on health promotion programs. Health Educ Q.

[53] Stokols D (1992). Establishing and maintaining healthy environments: toward a social ecology of health promotion. Am Psychol.

[54] Campbell C, Cornish F (2012). How can community health programmes build enabling environments for transformative communication? Experiences from India and South Africa. AIDS Behav.

[55] Proctor E, Silmere H, Raghavan R, Hovmand P, Aarons G, Bunger A, et al. Outcomes for implementation research: conceptual distinctions, measurement challenges, and research agenda. Adm Policy Ment Health Ment Health Serv Res. 2011;38. 10.1007/s10488-010-0319-7 PMC3068522 20957426

[56] Hubacher D, Trussell J (2015). A definition of modern contraceptive methods. Contraception..

[57] MEASURE Evaluation. Family planning and reproductive health indicators database: summary list of indicators. 2018. Retrieved from: https://www.measureevaluation.org/prh/rh_indicators/indicator-summary.

[58] Wegs C, Creanga AA, Galavotti C, Wamalwa E (2016). Community dialogue to shift social norms and enable family planning: an evaluation of the family planning results initiative in Kenya. PLoS One.

[59] Pulerwitz J, Barker G (2007). Measuring attitudes toward gender norms among young men in Brazil: development and psychometric evaluation of the GEM Scale. Men Masculinities.

[60] Shattuck D, Burke H, Ramirez C, Succop S, Costenbader B, Attafuah JD (2013). Using the inequitable gender norms scale and associated HIV risk behaviors among men at high risk for HIV in Ghana and Tanzania. Men Masculinities.

[61] Gottert AL. Gender norms, masculine gender-role strain, and HIV risk behaviors among men in rural South Africa: the University of North Carolina at Chapel Hill; 2014.

[62] Pulerwitz J, Hughes L, Mehta M, Kidanu A, Verani F, Tewolde S (2015). Changing gender norms and reducing intimate partner violence: results from a quasi-experimental intervention study with young men in Ethiopia. Am J Public Health.

[63] Nanda G. Compendium of gender scales. Washington, DC: FHI 360/C-Change 2011.

[64] Saldaña J (2015). The coding manual for qualitative researchers.

[65] Creswell J, Plano CV (2011). Designing and conduction mixed methods research. 2nd ed.

[66] Thabane L, Ma J, Chu R, Cheng J, Ismaila A, Rios LP (2010). A tutorial on pilot studies: the what, why and how. BMC Med Res Methodol.

[67] Lopez LM, Grey TW, Chen M, Hiller JE. Strategies for improving postpartum contraceptive use: evidence from non-randomized studies. Cochrane Database Syst Rev. 2014;(11):CD011298. 10.1002/14651858.CD011298.pub2 PMC11129846 25429714

[68] Lopez LM, Tolley EE, Grimes DA, Chen-Mok M. Theory-based interventions for contraception. Cochrane Database Syst Rev. 2011;3. 10.1002/14651858.CD007249.pub3 21412901

[69] Mwaikambo L, Speizer IS, Schurmann A, Morgan G, Fikree F (2011). What works in family planning interventions: a systematic review. Stud Fam Plan.

[70] Sonalkar S, Mody S, Gaffield ME (2014). Outreach and integration programs to promote family planning in the extended postpartum period. Int J Gynaecol Obstet.

[71] Ayanore MA, Pavlova M, Groot W (2016). Unmet reproductive health needs among women in some West African countries: a systematic review of outcome measures and determinants. Reprod Health.

[72] Sarkar A, Chandra-Mouli V, Jain K, Behera J, Mishra SK, Mehra S (2015). Community based reproductive health interventions for young married couples in resource-constrained settings: a systematic review. BMC Public Health.

[73] Steyn PS, Cordero JP, Gichangi P, Smit JA, Nkole T, Kiarie J (2016). Participatory approaches involving community and healthcare providers in family planning/contraceptive information and service provision: a scoping review. Reprod Health.

